# Computational thinking as a mediator between modeling self-efficacy and flexible thinking in pre-service science teachers

**DOI:** 10.3389/fpsyg.2026.1699956

**Published:** 2026-02-18

**Authors:** Rumeysa Beyazhancer, Salih Cepni

**Affiliations:** 1 Department of Mathematics Education, Faculty of Education, Bursa Uludağ University, Bursa, Türkiye; 2 Department of Science Education, Faculty of Education, Bursa Uludağ University, Bursa, Türkiye

**Keywords:** computational thinking, flexible thinking, mathematical modeling self-efficacy, mediation analysis, pre-service science teachers

## Abstract

**Introduction:**

In psychology, constructs such as self-efficacy, cognitive flexibility, and computational thinking (CT) are recognized as mechanisms that explain how individuals adapt to uncertainty and complexity. Self-efficacy regulates both motivation and cognition, while flexible thinking reflects the adaptive capacity to generate alternatives and cope with ambiguity. CT extends beyond technical domains as a cognitive framework that structures processes such as abstraction and reasoning. This study explores the mediating role of CT in the relationship between mathematical modeling self-efficacy and flexible thinking among pre-service science teachers.

**Methods:**

Participants were 244 pre-service science teachers from two state universities in Türkiye. Data were gathered using the Mathematical Modeling Self-Efficacy Scale, the Computational Thinking Skills Scale, and the Flexible Thinking in Learning Scale. Confirmatory factor analyses supported validity and reliability. PROCESS Macro (Model 4) tested mediation with controls for gender and grade.

**Results:**

Modeling self-efficacy significantly predicted CT (*β* = 0.375, *p <* 0.001), and CT significantly predicted flexible thinking (*β* = 0.368, *p <* 0.001). The direct effect of modeling self-efficacy on flexible thinking decreased from *β* = 0.512 to *β* = 0.378 when CT was included, indicating partial mediation. Bootstrap analysis confirmed the indirect effect (0.224, 95% CI [0.150, 0.298]).

**Discussion and conclusion:**

Findings indicate that self-efficacy beliefs influence adaptive cognitive capacities both directly and indirectly through CT. CT functions as a psychological bridge, linking self-efficacy with flexible thinking, and highlights the importance of integrating these constructs in educational psychology to foster resilience, adaptability, and coping with complexity.

## Introduction

Self-efficacy, flexible thinking, and computational thinking are conceptualized as cognitive–psychological constructs that reflect individuals’ beliefs and thinking processes involved in regulating action, adapting strategies, and solving complex problems in uncertain contexts (Bandura, 1997; De Corte, 2010; Wing, 2006), within the field of educational psychology. Flexible thinking, closely related to but broader than *cognitive flexibility*, refers to an individual’s capacity to shift perspectives, generate alternative strategies, and respond not only cognitively but also emotionally and socially to new situations (Barak and Levenberg, 2016a; Ionescu, 2012). Computational thinking (CT), originally conceptualized in computer science (Wing, 2006), has been increasingly recognized as a cognitive process of abstraction, decomposition, and adaptive reasoning with psychological implications beyond technical domains (Shute et al., 2017). Examining the dynamic interaction among these constructs offers valuable insights into resilience, creativity, and problem-solving capacity—skills that are essential in psychologically demanding environments.

Education has become a holistic process that aims to develop individuals’ multidimensional thinking skills through an interdisciplinary approach rather than solely focusing on knowledge transfer. Specifically, science teaching requires teachers to think multidimensionally and integrate different perspectives because it involves observation, inference, modeling, and problem-solving processes (Weintrop et al., 2016). Pre-service science teachers (PST) should be trained as individuals with high-level cognitive skills such as explaining natural phenomena, managing experimental processes, and interpreting scientific data. In this context, skills such as flexible thinking, CT, and mathematical modeling are critical in terms of providing an interdisciplinary perspective to science education. CT supports processes such as data analysis and algorithmic approaches in science teaching; thus, it enables pre-service teachers to present abstract scientific concepts in a more meaningful and structured way (Wing, 2006).

The integration of mathematical modeling into learning contexts provides valuable opportunities to enhance higher-order psychological processes such as scientific reasoning, problem solving, and adaptive thinking. Scientific reasoning—including hypothesis generation, evidence-based evaluation, and drawing conclusions—is a core component of learning (Kuhn, 2010; Zimmerman, 2000). From a psychological perspective, individuals’ confidence in their ability to apply mathematical modeling—modeling self-efficacy—shapes how they allocate cognitive resources, persist in problem solving, and cope with uncertainty (Bandura, 1997; Blomhøj and Jensen, 2003). At the same time, CT has been highlighted as a cognitive framework that links mathematical and scientific forms of reasoning (Grover and Pea, 2013; Wing, 2006; Chytas et al., 2024; Xu et al., 2022). CT components—such as decomposition, abstraction, pattern recognition, and algorithmic design—align closely with the cognitive demands of both modeling and flexible thinking (Weintrop et al., 2016). Rather than treating CT merely as a programming tool, recent scholarship argues for integrating CT concepts into scientific and mathematical thinking (Kafai and Proctor, 2022). In this study, we examine whether CT mediates the relationship between PSTs’ mathematical modeling self-efficacy and their flexible thinking, thereby clarifying a psychological pathway through which modeling-related beliefs may translate into broader adaptive thinking skills (Larkin, 2009; Veenman, 2015; Lye and Koh, 2014; Kwon et al., 2022).

CT is not merely a set of technical skills; it is a framework that enables the cognitive structuring of complex problems through abstraction, decomposition, pattern recognition, and algorithmic design (Wing, 2006; Weintrop et al., 2016; Shute et al., 2017). These processes closely align with indicators of flexible thinking, such as strategy switching, generating multiple solutions, and tolerance for ambiguity (Román-González et al., 2017; Lye and Koh, 2014; Kwon et al., 2022). Recent findings suggest that CT-based activities (coding/robotics, etc.) can support flexibility-related cognitive outcomes; however, the effect may be sensitive to task design (Montuori et al., 2024; Robledo-Castro et al., 2025). This theoretical and empirical framework suggests that CT may be not only a technical tool but also a mechanism that bridges adaptive cognition and flexible thinking. In this study, we address this gap by testing whether the aforementioned mechanism operates along the path of modeling self-efficacy → (CT) → flexible thinking.

**Figure 1:**
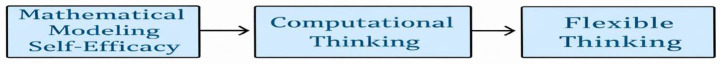
Conceptual framework.

Previous studies have examined the relationships between pre-service teachers’ mathematical modeling self-efficacy (H_1_), (CT) skills (H_2_), and flexible thinking (H_3_) from different perspectives and have generally reported significant and positive relationships between these variables (Koyuncu et al., 2017; Govender, 2020; Schukajlow et al., 2015). These findings indicate that these constructs play a critical role not only in terms of pedagogical competencies but also in terms of psychological processes such as cognitive flexibility, metacognitive strategy use, and problem-solving flexibility. However, the literature is quite limited in explaining how CT mediates the relationship between mathematical modeling self-efficacy and flexible thinking (H_4_). Yet, the core components of CT—abstraction, problem decomposition, pattern recognition, and algorithmic reasoning— overlap with the characteristics of flexible thinking, such as perspective shifting, generating multiple solutions, and coping with uncertainty (Wing, 2006; Larkin, 2009; Veenman, 2015; Kwon et al., 2022). Therefore, testing H_4_ fills an important gap in the literature by revealing the cognitive mechanism at work in transferring self-efficacy beliefs to flexible thinking. Figure 1 is presented to visualize a conceptual framework for empirically testing H_4_ regarding the mediating role of computational thinking; this framework serves to statistically examine the proposed mediation relationship.

The model illustrates the hypothesized relationships among three key psychological constructs: mathematical modeling self-efficacy, computational thinking, and flexible thinking. Mathematical modeling self-efficacy is expected to predict both computational thinking (H_1_) and flexible thinking (H_2_). Computational thinking is hypothesized to predict flexible thinking (H_3_) and to serve as a mediator in the relationship between modeling self-efficacy and flexible thinking (H_4_). This framework highlights the potential psychological mechanism through which self-efficacy beliefs in modeling may foster adaptive and flexible thinking skills.

### Mathematical modeling

Mathematical modeling and its curricular role have been examined from multiple perspectives, converging on the view that modeling involves transitions between real-world contexts and mathematical representations through a structured yet non-linear process. Modeling is commonly described as a cyclic activity, in which learners interpret situations, construct mathematical representations, and revise them through ongoing evaluation (Blum and Leiß, 2007). Research further shows that this process requires the coordinated use of cognitive and metacognitive strategies, including monitoring, decision-making, and regulation across modeling stages (Borromeo Ferri, 2006; Schoenfeld, 1985). Instructional frameworks such as Model-Eliciting Activities and process-focused approaches conceptualize modeling through sequential stages that allow movement back and forth rather than a strictly linear progression (Lesh and Doerr, 2003; Berry and Houston, 1995; Blum, 2011; Biccard and Wessels, 2011). Classroom-based and context-specific studies additionally indicate that difficulties in aligning authentic modeling tasks with curricular constraints often limit students’ engagement with the full modeling process (Mousoulides et al., 2006; Turner et al., 2022). Collectively, this literature portrays mathematical modeling as a complex activity that depends not only on cognitive competence but also on learners’ perceived self-efficacy in managing uncertainty and iterative decision-making (Bandura, 1997).

In this context, modeling self-efficacy, defined as beliefs about one’s capability to design and implement modeling activities, plays a critical role for pre-service science teachers (PSTs). Teachers with higher modeling self-efficacy are more likely to integrate modeling into inquiry-based instruction and to support authentic scientific practices (Schwarz et al., 2009; Windschitl et al., 2008; Oh, 2011; Passmore et al., 2009). Empirical studies have examined PSTs’ modeling self-efficacy (Koyuncu et al., 2017), documented their views and experiences (Ayvacı et al., 2015), and demonstrated its influence on pedagogical practices (Lingefjard, 2007; Erdoğan, 2019). Moreover, qualitative findings show that PSTs often struggle particularly with the stages of simplification and structuring in the modeling process, despite relative success in understanding problem contexts (Kenan and Polat, 2024; Deniz and Yıldırım, 2018). These findings underscore the need to strengthen PSTs’ modeling self-efficacy to support effective engagement with mathematical modeling.

#### Computational thinking

Computational thinking (CT) is an interdisciplinary competence that goes beyond computer science, involving problem-solving, modeling, and algorithmic reasoning (Wing, 2006; Denning, 2017; Weintrop et al., 2016). It enables the decomposition of complex problems into manageable parts and the development of step-by-step solutions. For teachers and pre-service teachers, CT is a key skill in technology-integrated instructional design, requiring not only technical knowledge but also pedagogical awareness to embed it effectively into students’ learning processes (Brennan and Resnick, 2012).

CT is gaining an increasingly important place in education. CT-based learning tools, coding environments, robotic kits, and interactive educational applications aim to ensure students’ active participation in the process. (Zhang and Nouri, 2019; Jiang and Li, 2021; Critten et al., 2022; Hooshyar et al., 2021). Indeed, it has been observed that students’ conceptual understanding and problem-solving skills have improved thanks to CT principles integrated into geometry (Hamid et al., 2022). Similarly, CT applications support students’ pattern recognition, situation analysis, and algorithm generation skills (Gadanidis et al., 2018; Rodríguez-Martínez et al., 2020).

Barr and Stephenson (2011) showed that CT in secondary education can be adapted to other disciplines such as mathematics, science, and social sciences. Furthermore, the researchers focused on the development of assessment tools to measure students’ CT skills. This has led to the creation of assessment tools specifically designed to measure students’ proficiency in areas such as algorithmic thinking and problem decomposition (Tang et al., 2020; Kang et al 2023). These assessments are important for determining the effectiveness of courses in developing students’ CT abilities.

CT skills from the early stages of teacher education equip PSTs to meaningfully and strategically integrate digital tools into their instructional practices (Yadav et al., 2014). CT is increasingly recognized as a fundamental competence not only for teachers but also for students, as it underpins problem-solving, analytical reasoning, and the capacity to approach complex tasks systematically (Wing, 2006; Grover and Pea, 2013; Camp, 2025). For teachers and pre-service teachers, CT supports the design of inquiry-oriented learning environments, facilitates the individualization of instruction, and enhances the ability to connect abstract concepts to real-world applications in mathematics and science (Kazakoff et al., 2013; Weintrop et al., 2016; Aldemir et al., 2025). For students, CT fosters higher-order cognitive skills, such as abstraction, modeling, and algorithmic thinking, which are essential for engaging with scientific and mathematical practices (Basu et al., 2013; Sengupta et al., 2013; Cheng et al., 2026; Başgül and Coştu, 2026; Lukitasari et al., 2025). Consequently, embedding CT-focused competencies into teacher education programmes and raising awareness of its pedagogical value are central to preparing educators who can cultivate these critical skills in their future learners.

#### Flexible thinking

Among the 21st century skills, flexible thinking is a fundamental competence that supports cognitive, emotional, and social development. It is defined as the ability to change or redefine information to adapt to new situations (Guilford, as cited in Barak and Levenberg, 2016a), enabling individuals to overcome thought fixity and generate creative solutions (Gocłowska et al., 2013; Li, 2020). Barak (2018) considers it a high-level cognitive skill essential in technology-enriched environments, while Ionescu (2012) emphasizes that flexible individuals adapt more successfully, self-manage, maintain emotional balance, show resilience, and lower neurotic tendencies. Deak (2000) defines it as a permanent mental structure allowing shifts in perspective, and McCrae (1987) together with Thurston and Runco (1999) highlight its role in changing strategies of interpreting and applying knowledge. The literature identifies three dimensions: cognitive (Martin and Rubin, 1995), emotional (Tugade and Fredrickson, 2004), and social (Martin and Anderson, 1998). Hall (1994) argue that thinking style outweighs genetics and intelligence, while Spiro and Jehng (1990), as cited in Barak and Levenberg (2016b) stress cognitive flexibility as a basic competence. Educationally, flexible thinking is critical for problem solving, knowledge transfer, and adapting to new learning contexts (Deak, 2000; Ionescu, 2012). In teacher education, it is essential for adapting strategies to diverse classrooms and fostering alternative solutions (Spiro et al., 1992; Martin and Rubin, 1995). Teachers with high cognitive flexibility better adjust pedagogy, integrate perspectives, and create inclusive learning environments (Dennis and Vander Wal, 2010; Zhang, 2017), and research shows that promoting flexible thinking enhances prospective teachers’ capacity to manage challenges and support students’ creative and critical thinking (Kuntze et al., 2017; Choi and Lee, 2009).

#### Mathematical modeling and computational thinking

Important stages of the mathematical modeling process are closely related to critical aspects of CT, since CT skills are directly applicable to modeling competencies (Ang and Tan, 2022). Kallia et al. (2021) examined the association of CT with mathematical modeling, showing how software tools that support students’ informatic skills enhance mathematics learning. Their framework illustrates two parallel processes: in mathematics, the cycle moves from “problem situation” to “mathematical model” and “mathematical solution” before reaching the “real solution”; in computer science, it progresses from “problem situation” to “computer science model,” “computer science solution,” and then to the “real solution.” Both highlight how real-world problems are represented within disciplinary structures and adapted back into practice, thus revealing the overlap of mathematical modeling and CT. Ang (2021) further demonstrated that modeling processes inherently activate CT components such as abstraction, decomposition, algorithmic thinking, and pattern recognition. Modeling tasks thus become a natural platform for the practice of CT, offering strong educational value when both are integrated.

#### Computational thinking and flexible thinking

CT provides a mental framework for analyzing complex problems (Wing, 2006), but it also requires individuals to shift strategies, adapt to unexpected situations, and generate new solutions. In this way, CT processes activate flexible thinking skills. Shute et al. (2017) emphasized that CT is not only technical but also supports cognitive flexibility. Ang (2021) argued that applying CT in modeling tasks triggers students’ flexible thinking capacities, while Montuori et al. (2024) found that coding and robotics activities improve children’s cognitive flexibility. Rheid Mohammed (2023) showed that cognitive flexibility mediates the relationship between CT and design thinking among IT students, and Robledo-Castro et al. (2025) reported mixed effects of CT training on flexibility, indicating that task design is crucial. Román-González et al. (2017) also highlighted that CT tasks promote strategy switching and multiple solutions, which are indicators of flexible thinking. Collectively, these findings suggest that CT both relies on and fosters flexible thinking, demonstrating their complementarity in complex problem solving.

#### Mathematical modeling and flexible thinking

Flexible thinking is the ability to restructure thought patterns, adopt different perspectives, and generate alternative solutions in the face of uncertainty (Gocłowska et al., 2013). Such adaptability is especially critical in open-ended problem situations. Mathematical modeling, which involves analyzing and solving real-world problems through mathematical representations, is one such context. Stillman (2011) stressed that modeling requires evaluating and revising models by shifting strategies when needed. Govender (2020) showed that modeling activities cultivate flexible thinking in pre-service teachers, enabling them to cope with uncertainty, explore different approaches, and adjust strategies. Similarly, Schukajlow et al. (2015) found that integrating alternative solutions into modeling tasks improved students’ problem-solving success and flexible thinking strategies, while Segura and Ferrando (2023) identified flexible strategies as critical for success in Fermi problems.

Research also shows that pre-service teachers with higher levels of modeling self-efficacy are better able to approach problems from multiple perspectives and modify strategies when faced with challenges (Kaiser and Stender, 2013; Schukajlow et al., 2018). Such adaptability aligns with flexible thinking, which involves shifting between cognitive frameworks and exploring alternative pathways (Spiro et al., 1992; Martin and Rubin, 1995). Mathematical modeling is therefore not only the application of mathematics but also a learning environment that fosters flexibility through iterative revisions, integration of diverse representations, and responsiveness to real-world constraints (Blum and Leiß, 2007; Borromeo Ferri, 2018).

In modern educational contexts, pre-service science teachers are expected to acquire higher-order thinking skills such as mathematical modeling, computational thinking (CT), and flexible thinking. CT, with its core components of decomposition, pattern recognition, abstraction, and algorithmic thinking, provides a powerful cognitive framework for science education. Its integration enables learners to use modeling and simulation to explain and investigate scientific phenomena. Prior research highlights CT’s close connection to disciplinary learning (Grover and Pea, 2013), its role in supporting agent-based modeling as a key aspect of scientific reasoning (Sengupta et al., 2013), and its importance in preparing students for contemporary practices in science, engineering, and technology (Aristawati et al., 2018; Lee et al., 2020). Collectively, these studies suggest that CT is not merely a technical skill but a way of thinking that fosters scientific problem solving, model construction, and authentic engagement in disciplinary practices. Embedding CT- and flexibility-oriented modeling tasks in science teacher education can therefore strengthen pre-service teachers’ modeling self-efficacy and enhance the overall quality of science education (Wing, 2006; Weintrop et al., 2016).

Despite these insights, the literature still lacks a comprehensive understanding of how modeling self-efficacy, CT, and flexible thinking interact with one another. In particular, few studies have investigated whether CT functions as a bridge between modeling self-efficacy and flexible thinking in the context of science teacher education. This gap limits the design of integrated instructional practices aimed at simultaneously developing cognitive and pedagogical competencies.

This study focuses on the relationships among pre-service science teachers’ mathematical modeling self-efficacy, computational thinking skills, and flexible thinking abilities. Specifically, it investigates whether computational thinking serves as a mediating variable in the relationship between modeling self-efficacy and flexible thinking—highlighting how these three skills interrelate within teacher education frameworks designed to support complex cognitive development.

**Figure 2:**
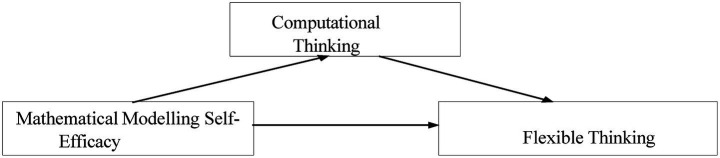
The research model.

This study investigates the mediating role of computational thinking (CT) in the relationship between pre-service science teachers’ mathematical modeling self-efficacy and flexible thinking. Using a relational survey design, it explores how modeling self-efficacy influences flexible thinking through CT as a cognitive mechanism. The findings are expected to inform holistic approaches to developing pre-service teachers’ instructional competencies. Based on this framework, the following hypotheses and the proposed research model (Figure 2) are presented.

*H_1_*: Mathematical modeling self-efficacy has a positive effect on computational thinking skills.

*H_2_*: Computational thinking skills have a positive effect on flexible thinking skills.

*H_3_*: Mathematical modeling self-efficacy has a positive effect on flexible thinking skills.

*H_4_*: Computational thinking skill mediates the relationship between mathematical modeling self-efficacy and flexible thinking skill.

## Materials and methods

### Design

This study employed a quantitative research design to examine the mediating role of computational thinking in the relationship between pre-service science teachers’ mathematical modeling self-efficacy and their flexible thinking skills. The selection of science teachers as the study sample was informed by the increasing emphasis on interdisciplinary and integrated STEM-oriented curricula, in which mathematical modeling functions as an important component for connecting scientific inquiry with computational thinking. Given that science teachers cannot consistently rely on direct collaboration with mathematics teachers in classroom practice, it has become essential for them to develop sufficient self-efficacy in mathematical modeling to design and implement modeling-based learning activities independently. Specifically, a correlational survey model was used to identify and analyze the relationships among the three variables. This design allows for the exploration of statistically significant associations without implying causality, which is particularly suitable for understanding naturally occurring patterns in educational research (Fraenkel and Wallen, 2011). In the proposed model, mathematical modeling self-efficacy served as the independent variable, flexible thinking as the dependent variable, and computational thinking as the mediating variable.

### Participants

The research study group consisted of 244 pre-service science teachers (PSTs) enrolled in science teaching programs at two state universities in the Marmara region of Turkey. Among them, 206 were female (84.4%) and 38 were male (15.6%), with a mean age of 20.7 years. Initially, data was collected from 256 teacher candidates for the study. During the data screening phase conducted prior to the analysis process, data from 12 participants who had incomplete responses, left a significant portion of the scales blank, or gave the same response to all items were excluded from the analysis. Accordingly, the final analyses of the study were conducted using a valid data set obtained from 244 participants. In the sample selection, convenience sampling, one of the purposive sampling methods, was preferred. This method allows for effective data collection by providing easy access to individuals with characteristics defined within the scope of the research (Fraenkel and Wallen, 2011). PSTs represent an important population for examining self-efficacy beliefs, cognitive adaptability, and flexible problem-solving capacities.

### Instrument and procedures

This research was conducted in accordance with ethical principles applicable to studies involving human participants. The research process was reviewed and approved by the relevant Social and Human Sciences Ethics Committee. Pre-service science teachers participating in the study were informed about the purpose, scope, and principles of voluntariness of the research; their informed consent was obtained. The participants’ identity information was kept confidential, and the data obtained were used solely for scientific purposes. The data collection tools used in the research consisted of self-report scales whose validity and reliability had been previously reported in the literature. The scales were administered face-to-face and under the supervision of the researchers at the schools where the pre-service science teachers were studying. Participants volunteered to take part in the study and were informed about the purpose and process of the research prior to the application. The average completion time for the questionnaires was approximately 18–20 min. Considering the total number of items in the scales used and the cognitive complexity of the structures measured, this time is considered sufficient for participants to carefully read and evaluate the items before responding. Before proceeding to the analysis process, the data were screened for suitability for statistical analysis. In this context, missing data, outliers, and response consistency were examined. Analyses were performed on the valid data set identified as a result of the preliminary review.

The Mathematical Modeling Self-Efficacy Scale (MMES) developed by Koyuncu et al. (2017) was used to determine the participants’ self-efficacy beliefs regarding mathematical modeling processes. The scale has a unidimensional structure and consists of 17 items. In the original study, the internal consistency coefficient of the scale was reported as *α* = 0.91. The internal consistency coefficient obtained in this study was *α* = 0.940. Participants used a five-point Likert-type response scale (1 = strongly disagree, 5 = strongly agree). CFA results: *χ*^2^/df = 1.76, CFI = 0.97, GFI = 0.95, AGFI = 0.94, RMSEA = 0.036.

In this study, the Computational Thinking Skills Scale (CTSS) developed by Korkmaz et al. (2017) was used to determine the computational thinking levels of pre-service teachers. The scale consists of a total of 22 items and is structured in five dimensions: creativity and algorithmic thinking, collaboration, critical thinking, and problem solving. In the original study, the overall internal consistency coefficient of the scale was reported as *α* = 0.86 and between 0.65 and 0.86 for the sub-dimensions. In this study, the scale was considered as a single-factor structure, and the internal consistency coefficient was found to be *α* = 0.760. Participants responded to the items on a five-point Likert-type scale ranging from 1 (strongly disagree) to 5 (strongly agree). CFA results are as follows: *χ*^2^/df = 2.14, CFI = 0.92, GFI = 0.91, AGFI = 0.89, RMSEA = 0.057.

Flexible Thinking in Learning Scale (FTLS), developed by Barak and Levenberg (2016a), and adapted into Turkish by Aktas et al. (2024), was used to determine the level of flexible thinking in the learning process. The scale originally consists of 19 items that pertain to three dimensions (open-mindedness, alternative generation, and awareness). In the original study, the internal consistency of the scale was reported as *α* = 0.91. In this study, the scale was considered a single-factor structure; the internal consistency coefficient was calculated as *α* = 0.848. The participants used a five-point Likert-type response scale (1 = strongly disagree, 5 = strongly agree). The fit indices obtained from the CFA analysis are as follows: *χ*^2^/df = 1.95, CFI = 0.94, G2I = 0.94, RMSEA = 0.051.

In this study, the mediating role of computational thinking skills in the relationship between mathematical modeling self-efficacy and flexible thinking was examined. Since it has been suggested that gender and grade level may have an effect on cognitive skills and self-efficacy beliefs, these two variables were included in the model as control variables. It is thought that gender may create differentiation, especially on psychological constructs such as thinking strategies and self-confidence, and grade level may affect self-efficacy and thinking skills of pre-service teachers in relation to the accumulation of knowledge and experience they have acquired in their educational processes.

The three scales used represent established psychological constructs, not merely as measurement tools in educational contexts. Accordingly, mathematical modeling self-efficacy is a domain-specific belief system based on Bandura (1997) self-efficacy theory; flexible thinking is considered a cognitive and behavioral adaptation capacity that goes beyond cognitive flexibility; and CT is considered a high-level cognitive structure consistent with metacognitive problem-solving processes such as abstraction, decomposition, and algorithmic reasoning.

### Data analysis

Data were analysed using SPSS 29.0 and PROCESS macro v4.1. Descriptive statistics for all study variables, including control variables (gender and grade level), are summarised in Table 1. Prior to hypothesis testing, univariate normality was assessed through skewness and kurtosis indices. All variables met approximate normality thresholds. To test the construct validity of the scales, a single-factor confirmatory factor analysis (CFA) was applied for each scale. Control variables were mean-centred to reduce multicollinearity and entered as covariates in all regression analyses. To test the mediating effect of CT (H_4_), PROCESS Model 4 (Hayes, 2018) was applied with 5,000 bias-corrected bootstrap samples (95% CI).

**Table 1:** Descriptive and correlation results of study variables.

Grade	*N*	Mean	SD	Skewness	Kurtosis	FT	MM	CT	Gender
FT	244	3.98	0.366	0.441	0.827	–			
MM	244	3.73	0.594	−0.146	0.855	0.499**	–		
CT	244	3.42	0.309	1.052	1.978	0.350**	0.515**	–	
Gender	244	1.16	0.363	1.911	1.664	−0.130*	0.098	0.138*	–
Grade	244	2.27	0.861	0.149	−0.668	0.058	0.109	0.027	0.102

FT, Flexible Thinking; MM, Mathematical Modeling Self-Efficacy; CT, Computational Thinking.

The symbols indicate levels of statistical significance: *p < 0.05; **p < 0.01.

### Research results

The table presents the descriptive statistics and correlation coefficients among the variables analysed in the study.

In order for the variables to have a normal distribution, the skewness and kurtosis values should be between −2 and +2 (George and Mallery, 2010). When Table 1 is examined, one can see that the calculated values are within the specified range and the assumption of normal distribution is met. The mean score for flexible thinking is 3.98 (SD = 0.366) 36. For mathematical modeling, the mean is 3.73 (SD = 0.594). The mean score for computational thinking is 3.42 (SD = 0.309). There is a strong positive correlation between flexible thinking and mathematical modeling (r = 0.499, *p <* 0.01). A moderate positive correlation exists between computational thinking and flexible thinking (r = 0.350, *p <* 0.01). Additionally, a strong positive correlation is found between computational thinking and mathematical modeling (.r = 0.515, *p <* 0.01). According to the results of the correlation analysis, significant positive relationships were observed between flexible thinking and mathematical modeling; computational thinking and flexible thinking; and computational thinking and mathematical modeling. After determining these significant relationships between the variables, the analysis of the mediating effects was conducted.

### Analyses related to hypotheses

In line with the hypotheses of the study, the results of the mediation analysis obtained with Process Macro are given in Table 2. This analysis determines the relationship between pre-service teachers’ knowledge, mathematical modeling self-efficacy, and computational thinking; and between flexible thinking skills; mathematical modeling self-efficacy and flexible thinking skills; and the mediating role of computational thinking skills in the relationship between mathematical modeling self-efficacy and flexible thinking skills, which is the main purpose of our research, are given in Table 2. To test a possible mediating role of computational thinking, regression-based mediation analyses were conducted using procedures provided by Hayes (2018).

**Table 2:** Analysis of mediating effects of computational thinking.

Variables		Overall fit index			95% CI				
Result	Predictor	*R*	*R* ^2^	*F*	*β*	LLCI	ULCI	*t*	*p*
FT	Gender	0.529	0.279	31.103**	0.159	0.081	0.439	2.863*	< 0.001
	Grade				0.062	−0.031	0.118	1.135	> 0.001
	MM				0.512	0.660	1.011	9.316*	< 0.001
CT	Gender	0.396	0.157	14.897**	0.188	0.059	0.261	3.143*	< 0.001
	Grade				−0.013	−0.047	0.037	−0.228	> 0.001
	MM				0.375	0.217	0.417	6.261*	< 0.001
FT	Gender	0.627	0.394	38.890**	0.089	−0.021	0.314	1.722	> 0.001
	Grade				−0.067	−0.022	0.115	1.334	> 0.001
	MM				0.378	0.437	0.788	6.882	< 0.001
	CT				0.368	0.437	0.787	6.716*	< 0.001

*p < 0.05, **p < 0.01, ***p < 0.001. CT, Computational Thinking; MM, Mathematical Modeling Self-Efficacy; FT, Flexible Thinking.

The gender and grade variables of pre-service science teachers were included in the analysis as control variables, and the mediating effect of computational thinking on the relationship between flexible thinking and mathematical modeling self-efficacy was analyzed. In the multiple regression analysis conducted to test the mediation effect of computational thinking skill between flexible thinking and mathematical modeling self-efficacy, PROCESS was used as the mediation model (Model 4). According to the results of Table 2, mathematical modeling self-efficacy has a significant and positive effect on computational thinking skills (*β* = 0.375, *p <* 0.001; 95% CI [0.217; 0.417]). This supports hypothesis 1. Computational thinking has a strong and significant effect on flexible thinking ability (*β* = 0.368, *p <* 0.001; 95% CI [0.437; 0.787]). The direct effect was identified as the effect of mathematical modeling self-efficacy on flexible thinking (*β* = 0.512, *p <* 0.001; 95% CI [0.660; 1.011]). These results support the validity of hypothesis 2 and hypothesis 3. Based on these results, hypotheses H_1_, H_2_ and H_3_ are accepted.

**Figure 3:**
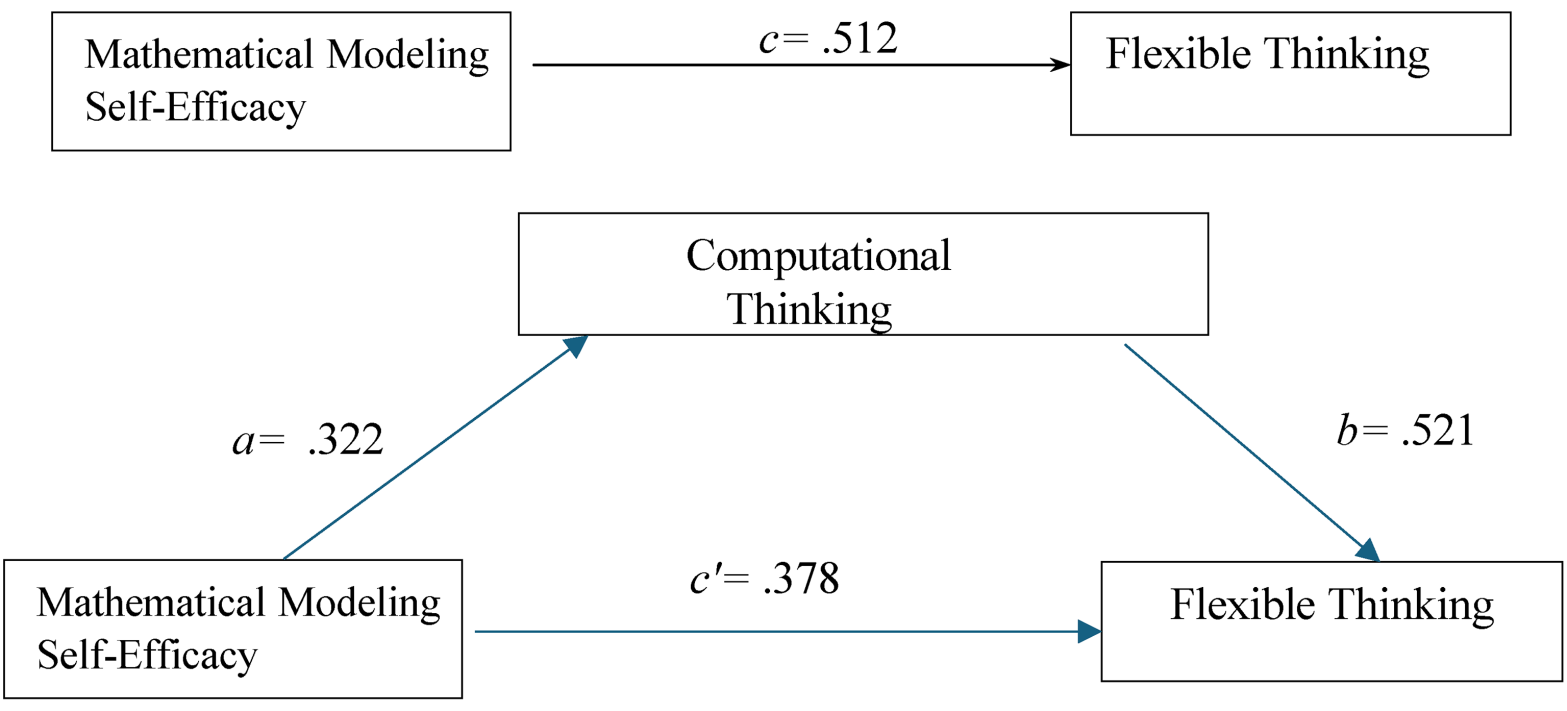
The mediating role of computational thinking in the relationship between mathematical modeling self-efficacy and cognitive flexibility.

According to the results of the analysis, mathematical modeling self-efficacy continued to have a positive and statistically significant effect on flexible thinking after the inclusion of computational thinking as a mediator (*β* = 0.378, *p <* 0.001; 95% CI [0.437; 0.788]). When the standardized regression coefficients were examined, the effect of mathematical modeling self-efficacy on flexible thinking decreased from *β* = 0.512 (when entered as the sole predictor) to *β* = 0.378 after computational thinking was included in the model. This reduction in the magnitude of the direct effect, while remaining statistically significant, indicates the presence of a partial mediation effect. Furthermore, the mediation analysis revealed that computational thinking significantly mediated the relationship between mathematical modeling self-efficacy and flexible thinking, with a significant indirect effect (indirect effect = 0.2242). In other words, computational thinking plays a significant and partial mediating role in the relationship between mathematical modeling self-efficacy and flexible thinking. Thus, H4 is supported. The result of the mediation model is presented in Figure 3.

## Discussion

This study aimed to examine the mediating role of CT in the relationship between pre-service science teachers’ mathematical modelling self-efficacy and flexible thinking skills. The results revealed significant positive relationships among all three variables, supporting the proposed model and confirming all four hypotheses. Specifically, pre-service teachers’ mathematical modelling self-efficacy was a significant predictor of CT (*β* = 0.375, *p <* 0.001), and CT significantly predicted flexible thinking (*β* = 0.368, *p <* 0.001). Moreover, mediation analysis demonstrated that CT partially mediated the effect of mathematical modelling self-efficacy on flexible thinking (indirect effect = 0.224, 95% CI [0.150; 0.298], *p <* 0.001). These findings suggest that pre-service teachers’ confidence in modelling processes may not only directly support flexible thinking but also indirectly through the enhancement of CT skills. This result is consistent with Bandura’s (1997) conceptualization of self-efficacy as a belief system that regulates motivation and cognition, suggesting that modelling self-efficacy shapes adaptive thinking via cognitive mechanisms.

The strong link observed between mathematical modelling self-efficacy and flexible thinking is supported by previous studies highlighting the cognitive demands of modelling activities. Govender (2020) conceptualized mathematical modelling as a process that fosters flexible thinking and creativity by requiring learners to work with uncertainty, generate alternative strategies, and adapt their reasoning. Similarly, Schukajlow et al. (2015) emphasized that open-ended modelling problems promote strategic diversity, while Segura and Ferrando (2023) found that flexible strategies are key predictors of success in mathematical problem solving. These findings support the view that modelling is not only a mathematical task but also a learning environment that can strengthen mental flexibility, aligning with broader definitions of flexibility (Gocłowska et al., 2013; Ionescu, 2012). Thus, mathematical modeling self-efficacy is not merely a task-specific competence but, as noted in the literature (Bandura, 1997; Ionescu, 2012), may also reflect a belief system that is associated with individuals’ psychological resilience and adaptability when facing complex and uncertain problems.

In addition, this study confirmed that mathematical modelling self-efficacy predicted CT, as evidenced by the significant regression coefficient (*β* = 0.375, *p <* 0.001), reflecting the computational nature of modelling tasks. Wing (2006) and Brennan and Resnick (2012) identified abstraction, decomposition, and algorithmic design as foundational CT skills that are inherently embedded in the modelling process. As learners transition between real-world contexts and mathematical representations, they apply recursive reasoning and systematized procedures—hallmarks of computational thinking (Ang, 2021). This indicates that modelling competence involves more than content knowledge; it requires algorithmic fluency and problem structuring, which are central to CT development. From a psychological standpoint, CT here functions as an adaptive cognitive tool, enabling individuals to externalize and regulate thought processes that underpin flexible reasoning.

The observed relationship between CT and flexible thinking further supports the view that CT enhances not only technical skills but also cognitive flexibility. Shute et al. (2017) and Barak (2018) argued that CT fosters adaptive behavior, as it challenges learners to persist through failure, shift strategies, and tolerate ambiguity—essential characteristics of flexible thinkers. Montuori et al. (2024) showed that robotics and coding applications improve students’ ability to generate flexible strategies and diverse solutions, while Román-González et al. (2017) and Rodríguez-Martínez et al. (2020) demonstrated that CT is positively associated with higher-order reasoning and adaptability. These findings are consistent with the view that CT is not just a computational skill set but also a cognitive resource that contributes to creative and resilient thinking. In psychological terms, CT can be seen as a mediator that transforms self-efficacy beliefs into adaptive coping strategies.

The partial mediation result observed in this study suggests that CT functions as a cognitive bridge linking modelling self-efficacy and flexible thinking. While a substantial portion of the effect of mathematical modelling self-efficacy on flexible thinking is direct, the indirect pathway through CT indicates that fostering CT skills may strengthen the transfer of modelling competence into broader cognitive adaptability. This interpretation is conceptually consistent with the findings of Ang (2021), Kallia et al. (2021), and Stillman (2019), who emphasized the structural parallels between modelling and CT and proposed integrated frameworks for their development. This mechanism suggests that self-efficacy beliefs may not exert their influence on complex psychological outcomes (such as flexibility) solely in a direct way, but rather through cognitive mediators such as CT, a view that aligns with prior accounts highlighting the indirect pathways of self-efficacy (e.g., Schunk and Pajares, 2005).

However, the partial nature of the mediation also implies that other variables may contribute to this relationship. Factors such as epistemic beliefs, motivation (Zhang and Nouri, 2019), digital experience, and emotional flexibility (Tugade and Fredrickson, 2004) may also play influential roles. Moreover, the cross-sectional design limits causal interpretations, suggesting that future research should employ longitudinal or experimental designs to further validate the findings.

From an educational perspective, these results underscore the importance of designing instruction that simultaneously cultivates CT, flexible thinking, and modelling competencies. Previous work by Yadav et al. (2014) and Grover and Pea (2013) has shown that teachers equipped with CT and flexible thinking are more capable of scaffolding complex reasoning and adapting to diverse learning contexts. Including modelling-based, problem-driven activities that foster algorithmic reasoning and cognitive flexibility may enhance teacher candidates’ readiness for real-world teaching challenges.

Finally, it is important to view flexible thinking not merely as a personality trait but as a performance-based skill that can be cultivated through targeted instructional strategies. As argued by Deak (2000) and Barak and Levenberg (2016a), cognitive flexibility emerges in response to rich, uncertain, and meaningful learning environments—precisely the kind offered by integrated modelling and CT tasks.

## Conclusions and implications

This study provides empirical evidence for the partial mediating role of computational thinking (CT) in the relationship between pre-service science teachers’ mathematical modelling self-efficacy and their flexible thinking skills. The results suggest that CT functions not only as an independent cognitive skill but also as a facilitating mechanism that enhances the transfer of modelling confidence into adaptive thinking strategies, with important psychological implications for self-efficacy and cognitive adaptability.

The findings underscore the importance of equipping teacher candidates with both modelling skills and CT abilities in order to foster their flexible thinking. This trio of competencies— mathematical modelling self-efficacy, CT, and flexible thinking—represents not only pedagogical tools but also psychological constructs that support resilience, motivation, and adaptive problem solving. The study stresses that flexible thinking is not a fixed trait but a learnable skill that can be cultivated through meaningful modelling and problem-solving activities grounded in computational processes.

In the present study, all variables were measured simultaneously using self-report instruments. This methodological choice raises the possibility of common method bias. To mitigate this potential effect, data collection was conducted face-to-face under researcher supervision, and participants were informed that their responses would remain confidential and be used solely for scientific purposes. Nevertheless, it is acknowledged that common method bias cannot be entirely eliminated. Therefore, the findings should be interpreted with caution, taking into account the self-report nature of the measures and the cross-sectional design of the study.

From a practical standpoint, teacher education curricula should integrate instructional designs that target these interconnected skills. For example, including open-ended modelling tasks, CT-based coding environments, and problem-based learning scenarios could help foster not only modelling fluency but also the ability to flexibly approach complex teaching situations. In this sense, the implications extend beyond pedagogy to the psychological preparation of future teachers for uncertainty and complexity in classroom contexts.

In conclusion, this study emphasizes that modelling self-efficacy, when combined with computational competence, lays the groundwork for cognitive flexibility. Supporting pre-service teachers in developing these intertwined skills is essential not only for effective instruction but also for their psychological readiness to cope with the demands of modern educational environments.

## Data Availability

The raw data supporting the conclusions of this article will be made available by the authors, without undue reservation.
